# Dissolution of Microcrystalline Cellulose in Phosphoric Acid—Molecular Changes and Kinetics

**DOI:** 10.3390/molecules14125027

**Published:** 2009-12-04

**Authors:** Junhua Zhang, Jingqiang Zhang, Lu Lin, Tianming Chen, Jun Zhang, Shijie Liu, Zhenjiang Li, Pingkai Ouyang

**Affiliations:** 1 Department of Resources Science and Engineering, State Key Laboratory of Pulp and Paper Engineering, South China University of Technology, Guangzhou 510640, Guangdong Province, China; 2 Department of Paper and Bioprocess Engineering, College of Environmental Science and Forestry, State University of New York, 1 Forestry Drive, Syracuse, NY 13210, USA; 3 College of Medicine and Life Science, Nanjing University of Technology, Nanjing, 210009, China

**Keywords:** kinetic, MCC, phosphoric acid, decrystallization, structural change

## Abstract

In this study, we aimed to dissolve microcrystalline cellulose (MCC) with phosphoric acid to obtain high-quality fermentable saccharides. MCC was directly dissolved in phosphoric acid (the concentration was 83%) for 10 hours at temperatures of 30, 50, and 70 °C. The structural changes of MCC were determined in detail with X-ray powder diffraction, solid-state cross-polarization magic angle spinning ^13^C-NMR, and X-ray photoelectron spectroscopy. The kinetics of MCC decrystallization during treatment with phosphoric acid was also compared at 30, 50, and 70 °C. With the assumption of first order kinetics, the Arrhenius parameters of *K*, *A_0_* and *E_a_* were calculated. The rate constants of decrystallization reaction (*K*) were 0.06, 0.17, and 0.12 h^-1^ respectively. The pre-exponential factor (*A_0_*) was 1.2 × 10^6^ h^-1^, and the activation energy (*E_a_*) was 42.4 k J/mol.

## 1. Introduction

It is well known that petrochemical resources are diminishing and alternatives must be found to produce the energy and chemical materials required by society. In order to meet the growing demand for energy, microcrystalline cellulose (MCC) can serve as a sustainable source of renewable fuels and chemicals.

Cellulose is the most abundant renewable polymer which can be derived from plant biomass, and its efficacious utilization would represent a significant source of sustainable energy. However, due to its compact crystalline structure which is formed mainly by inter- and intra-molecular hydrogen bonds, cellulose is usually difficult to hydrolyze into fermentable sugars [1]. Therefore, this highly-ordered cellulose structure is very difficult to dissolve with chemicals or bio-enzymes [2,3,4,5,6], and this rigidity poses a challenge to the efficacious utilization of cellulose. Thus, a better method for the decrystallization of lignocellulose is urgently needed for improving its utilization efficiency and producing simple sugars for fermentation to produce ethanol fuel and other bio-based products [7]. 

The traditional decrystallization pathways for MCC include using physical [8,9,10,11] or chemical methods. The chemical methods include the use of ionic liquids [12,13], NaOH/urea [14] or phosphoric acid [15,16,17,18]. In the chemical method, phosphoric acid has been the most popular solvent for dissolution of crystalline cellulose for over 80 years due to its non-corrosive and nontoxic properties, its safe use and low cost compared to other inorganic mineral acids [19]. Walseth [20] first developed a procedure for producing high-reactivity cellulose suitable for cellulose activity studies by swelling air-dried cellulose in 85% phosphoric acid. After dissolving crystalline cellulose, the solubilized precursors can be formed, which can subsequently be catalytically hydrolyzed, using either biological or synthetic catalysts. Cellulose dissolution in phosphoric acid involves two main processes: 

(1) An esterification reaction between hydroxyl groups of cellulose and phosphoric acid to form cellulose phosphate:

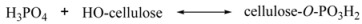

and (2) a competition of hydrogen-bond formation between hydroxyl groups of cellulose chains and hydrogen-bond formation between one hydroxyl group of a cellulose chain and a water molecule or with a hydrogen ion Meanwhile, another by-reaction, acid hydrolysis of β-glucosidic bonds of cellulose will take place. However, such acid hydrolysis can be minimized by decreasing the dissolution temperature [21]. During the regeneration process by water, cellulose phosphate reversibly can be converted back to free phosphoric acid and amorphous cellulose without any significant substitution or recrystallization.

In this study, MCC was dissolved in phosphoric acid (83%) at different temperatures. The structural changes of cellulose were analyzed by X-ray powder diffraction (XRD), Solid-State Cross-Polarization Magic Angle Spinning (CP/MAS) ^13^C-NMR spectroscopy and X-ray photoelectron spectroscopy (XPS). Our aim was to reduce the crystallinity of MCC cellulose by treatment with phosphoric acid in order to obtain under mild hydrolysis conditions high-quality fermentable saccharides which can be converted into bio-ethanol or bio-based chemicals.

## 2. Result and Discussion

### 2.1. The χc change of MCC analyzed by XRD

**Figure 1:**
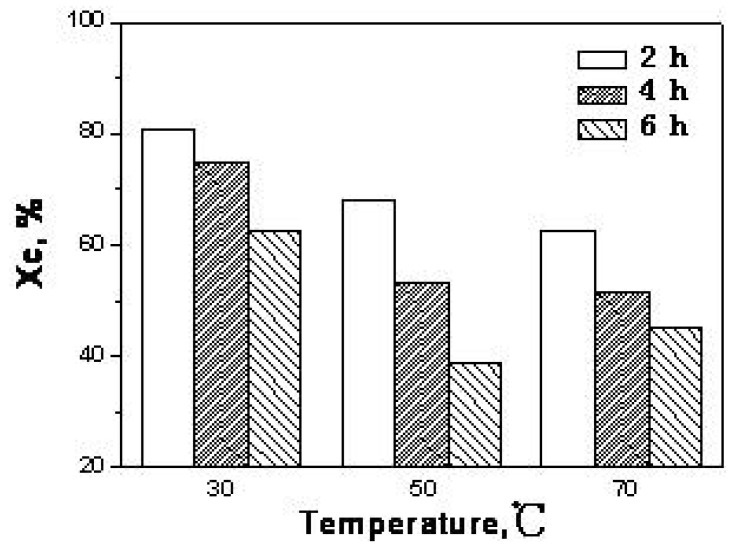
Effect of temperature on decrystallization of MCC with phosphoric acid.

Figure 1 shows the effect of temperature on the decrystallization of MCC with phosphoric acid at 2, 4, and 6 h. At 30 °C, the *χ_c_* numbers were 79.4%, 72.6%, and 63.1%, respectively, for 2, 4, and 6 h. At 50 °C, the *χ_c_* numbers were decreased to 70.6%, 57.3%, and 39.1%, respectively, for 2, 4, and 6 h, then to 63.8%, 50.6%, and 43.4% at 70 °C. At temperatures of 30 °C and 50 °C, the degrees of crystallinity of the cellulose decreased with increasing reaction time, which is in agreement with the reports of Ekenstam *et al*. [22] and Danilove *et al*. [23]. However, at the higher temperature of 70 °C, with a longer reaction time (for example, 6 h in this study), the decrystallization effect was reduced, which is undesirable because cellulose of low crystallinity is easier to hydrolyze. 

**Figure 2:**
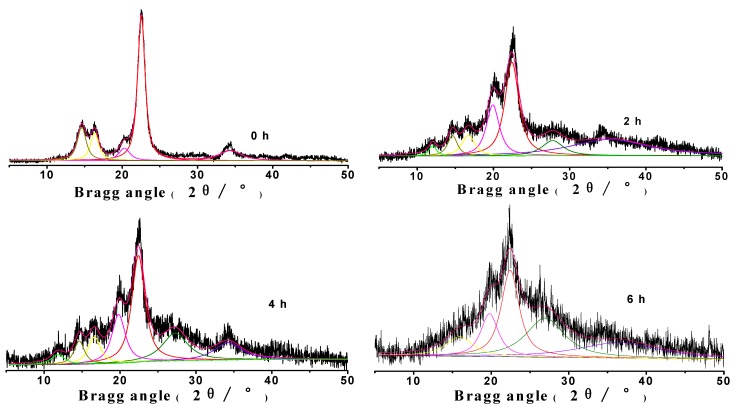
X-ray diffraction patterns of MCC samples with phosphoric acid at 50 °C. The characteristic peaks of cellulose I were: 2θ = 14.7°, 16.5°, 22.8°, and 34.5°; The characteristic peaks of cellulose II were: 2θ =12° and 20°.

Figure 2 shows the XRD patterns of MCC treated with phosphoric acid at 50 °C at different times. The MCC spectrum showed characteristic peaks of cellulose I at the 2θ of 14.7°, 16.5°, 22.8°, 34.5°, and cellulose II at the 2θ of 12°, 20°, respectively. With the dissolution of cellulose, the crystal peaks decreased greatly, and the degree of crystallinity also decreased from 92.2% to 39.1%. Thus, the regenerated cellulose prepared with phosphoric acid had low crystallinity and the reaction with phosphoric acid had markedly destroyed the crystal regions of the MCC. The relative content of amorphous regions increased. It can also be seen that the breadth of the characteristic cellulose II 2θ peak at 20° increased when MCC treated with phosphoric acid for 2 hours, and then, decreased gradually. Moreover, this characteristic 2θ peak at 12° begun to appear with the treatment of phosphoric acid, and then disappeared at 6 h, all of these indicating that the cellulose II was a transition form during the course of the transformation of cellulose I into amorphous cellulose.

### 2.2. CP/MAS ^13^C-NMR Analysis

The chemical shifts detected with CP/MAS ^13^C solid-state NMR spectra of the carbons (C_1_–C_6_) in MCC are shown in Table 1. 

**Table 1:** Resonance assignments for the CP/MAS ^13^C NMR spectra of cellulose and cellulose treated using phosphoric acid.

Carbon atom	Chemical shift ( ppm, δ)		
	Xc = 92.23%	Xc = 71.73%	Xc = 67.47%
C_1_	106.7,106, 105	106.8，106.2，105.3	106.8，105.9，105.3
Crystalline C_4_	89.9	89.9	90
Amorphous C_4_	84.6	83.7	83.6
C_2_, C_3_, C_5_	75.8, 73.4, 72.3	76, 75.5, 73.7	76.1, 73.7, 72.6
Crystalline C_6_	65.9	66.5	66.1
Amorphous C_6_	63.5	64.8	63.3

**Figure 3:**
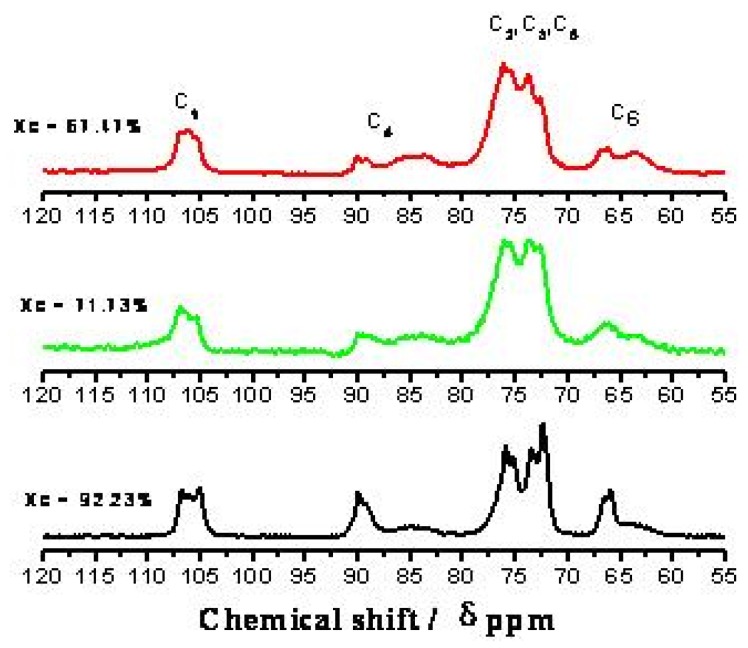
CP/MAS ^13^C-NMR spectra of MCC. The labeled peaks (labels are above the MCC trace) represent the intensities of carbons 1-6 (C_1_-C_6_).

Figure 3 shows that the cellulose C_1_ of MCC has three characteristic peaks at 105 ppm. The cellulose C_4_ has two characteristic peaks at 80~92 ppm. The cellulose C_6_ has two characteristic peaks at 58~69 ppm. MCC has three characteristic peaks at 72~78 ppm which are the characteristic peaks of the cellulose C_2_, C_3_ and C_5_, but there is disagreement about the assignment of the signals of these carbons. Teeäär *et al*. [24] thought that the signals at 76.8 and 76.0 ppm could be ascribed to the cellulose C_2_, and the one at 73.0 ppm was attributable to cellulose C_5_, and the intensity of 74.2 ppm was assigned to the cellulose C_3_, but Kono *et al*. [25] deemed that the signals at 76.8 and 76.0 ppm were from the cellulose C_3 _and Bardet *et al*. [26] considered that the 76.0 ppm signal corresponded to cellulose C_2_, and the peak at 74 ppm was from the cellulose C_3_ and C_5_. For all of these reasons, we did not discuss the characteristic peaks of C_2_, C_3_ and C_5 _in this paper.

**Figure 4:**
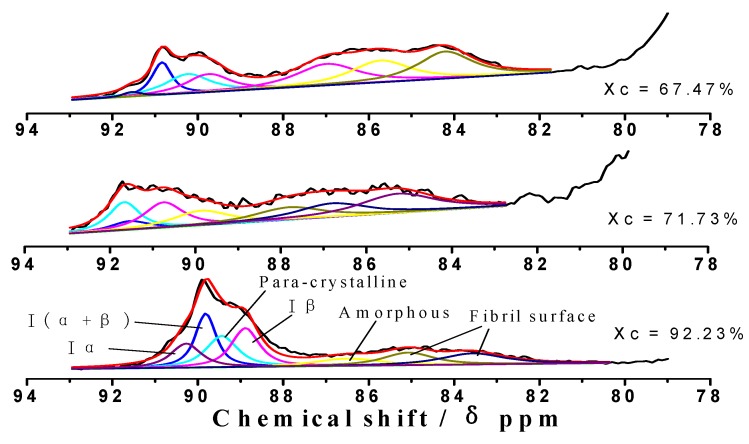
Results of fitting the C_4_ region of CP/MAS ^13^C-NMR spectra from MCC with different degrees of crystalline structure: The proportions of cellulose Ia, I(a + β), para-crystalline, and Iβ are identified in the first peaks; the proportions of amorphous cellulose and fibril surfaces are identified in the second peaks.

In the spectra of CP/MAS ^13^C solid-state NMR, the most valuable peak was C_4_. It was considered that the peak at 80~86 ppm was from the amorphous zone, and the peak 86~92 ppm was the crystalline and para-crystalline zones [27,28]. Figure 4 and Table 2 shows the distinct steps of C_4_ in the conversion from the crystalline zone to an amorphous zone with phosphoric acid treatment [29,30,31]. 

**Table 2:** Assignments and Intensity of Non-linear Least-squares fitting of the C_4_ region of the CP/MAS ^13^C-NMR Spectra from MCC with different degrees of crystallinity.

Xc, %	Chemical shift Assignments	Chemical shift ppm	Intensity %
92.23	Іα	90.3	12.6
	І (α+β)	89.8	19.6
	Para-crystalline	89.4	17.3
	Іβ	88.9	19.0
	Amorphous	86.5	8.2
	Fibril surface	85.1	11.1
	Fibril surface	83.6	12.2
71.73	Іα	89.7	5.2
	І (α+β)	89.9	13.8
	Para-crystalline	88.9	16.0
	Іβ	88.1	13.1
	Amorphous	86	13.5
	Fibril surface	85	15.3
	Fibril surface	83.5	23.1
67.47	Іα	90.7	1.3
	І (α+β)	90.1	9.8
	Para-crystalline	89.4	13.3
	Іβ	89	12.3
	Amorphous	86.2	20.6
	Fibril surface	84.9	19.9
	Fibril surface	83.4	22.8

With the decrease of the degree of crystallinity, the percentages of Іα, І(α+β), para-crystaline, and Іβ forms of crystalline cellulose C_4_ are greatly reduced from 68.5% to 48.1% and to 36.7%, respectively. The corresponding amorphous cellulose C_4_ increased from 31.5% to 51.9% and to 63.3%, respectively. 

**Figure 5:**
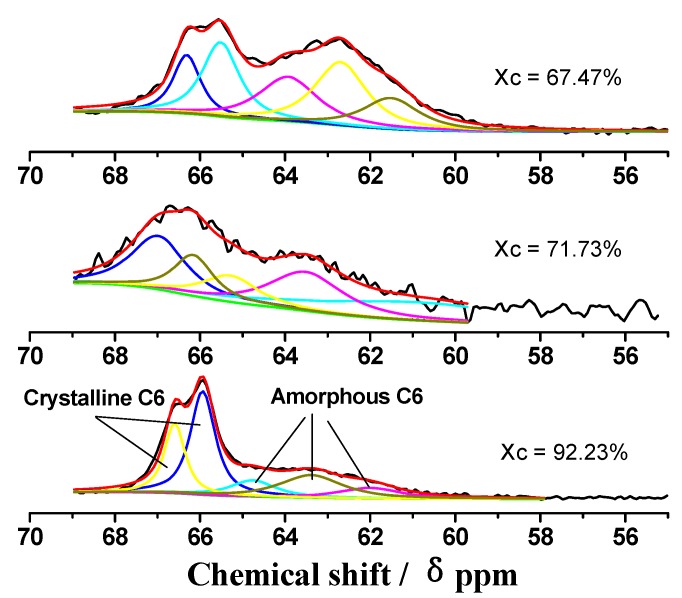
Results of fitting the C_6_ region of CP/MAS ^13^C-NMR spectra from MCC with different degrees of crystalline structure: The proportions of crystalline cellulose are identified in the first peaks; the proportions of amorphous cellulose are identified in the second peaks.

Figure 5 and Table 3 shows the distinct steps of C_6_ in the conversion from the crystalline zone to an amorphous zone with phosphoric acid treatment. With the decreasing of degree of crystallinity, the percentages of Іα, І(α+β), para-crystaline, and Іβ forms of crystalline cellulose C_6_ are greatly reduced from 59.4% to 35.4% and to 32.4%, respectively. The corresponding amorphous cellulose C_6_ increased from 40.6% to 64.6% and to 67.6%, respectively. All these results indicated that the compact network structure of cellulose became looser with the rupture of hydrogen bonds as a result of the step by step decrystallization process induced by phosphoric acid.

**Table 3:** Assignments and Intensity of Non-linear Least-squares fitting of the C_6_ region of the CP/MAS ^13^C-NMR Spectra from MCC with different crystalline degree.

Xc, %	Chemical shift Assignments	Crystalline C_6_	Amorphous C_6_
92.23	Chemical shift (ppm)	66.6，65.9	64.8，63.4，62
	Intensity (%)	21.4，38	10.8，20.4，9.4
	Total Intensity (%)	59.4	40.6
71.73	Chemical shift (ppm)	66.8，65.9	65.1，63.3，60.7
	Intensity (%)	22.7，12.7	11.3，22.3，30.9
	Total Intensity (%)	35.4	64.6
67.47	Chemical shift (ppm)	66.9，66.1	64.5，63.3，62.1
	Intensity (%)	13.4，18.9	23.7，29.3，14.6
	Total Intensity (%)	32.4	67.6

### 2.3. XPS analysis of MCC treated with phosphoric acid

**Figure 6:**
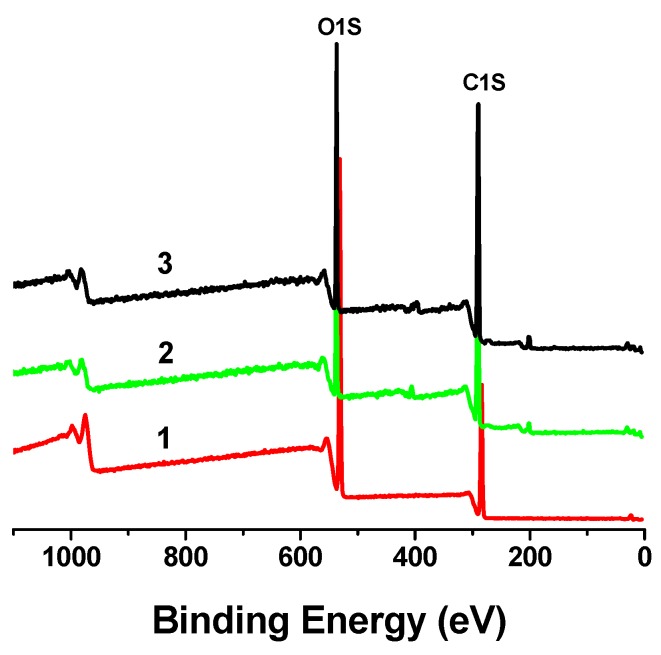
XPS wide scan patterns of MCC with different crystalline degree shows the O_1S_, N_1S_, and C_1S_ binding energies: Sample 1 (Xc = 92.23%); Sample 2 (Xc = 71.73%); Sample 3 (Xc = 67.47%).

Figure 6 shows the XPS wide scan spectra of MCC. There are only two significant peaks: O_1S_ and C_1S_, exist in the untreated MCC (sample 1). However, after the decrystallization treatment, a weak peak could be detected at around 401 eV, which is the signal for N_1S_ (the spectra of sample 2 and 3). This result implies that a very small amount of nitrogen existed on the surface of cellulose, even after careful washing. In the crystalline structure of cellulose, there are two kinds of hydrogen bonds: (1) O–H···O and (2) C–H···O, which are closely related to the oxygen and carbon atoms in the cellulose macromolecule.

**Figure 7:**
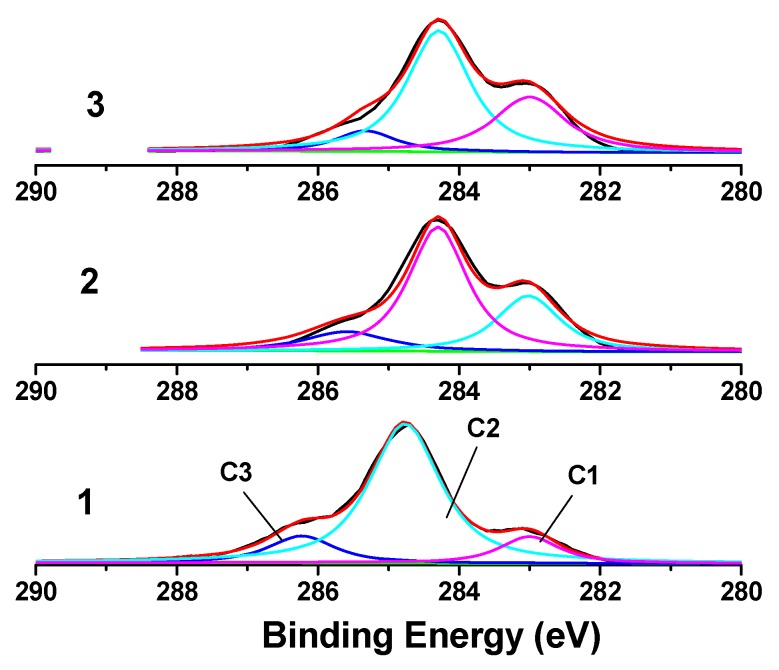
C_1s_ XPS patterns of MCC with different crystalline degree show the three phases of C_1S_: Sample 1 (Xc = 92.23%); Sample 2 (Xc = 71.73%); Sample 3 (Xc = 67.47%).

In the XPS spectra of cellulose, C_1S_ can be divided into three phases [32]: (1) C_1_ is the carbon atom linking only with a carbon or hydrogen atom at low binding energy (at about 283.00 eV), forming the chemical bond C-C or C-H; (2) C_2_ is the carbon atom linking with one oxygen atom not from a carbonyl group, but from a hydroxyl (-OH) group; binding energy is about 284.77 eV, forming the chemical bond C-OH; (3) C_3_ is the carbon atom linking with two oxygen atoms not from a carbonyl group, or with one oxygen atom of a carbonyl group, forming the chemical bond O-C-O or C=O, with a binding energy of about 286.23 eV. Figure 7 shows the C_1S_ XPS spectrum of MCC samples of different degrees of crystallinity. The C_1_ (C-C/C-H) contents are 14.5%, 28.2%, and 33.1%, respectively, for sample 1, 2, and 3. The C_2_ (C-OH) are 68.1%, 58.9%, and 56.3%, respectively, and for C_3 _(O-C-O) are 16.4%, 12.9%, and 10.6%, respectively. These data have been calculated from the corresponding relative peak areas and the C_1_ (C-C/C-H) and C_2_ (C-OH) peaks are the predominant ones. It is well known that there are three active hydroxyls (-OH) on the No. 2, 3, 6 carbons of the β-D-glucopyranose unit of cellulose, forming intramolecular hydrogen bonds C_3_-OH…O_5_, C_2_-OH…O_6_ and the intermolecular hydrogen bond C_6_-OH…O_3_. This indicates that a hydroxyl (-OH) domain exists in the inner cellulose structure and the units of lignin and hemicellulose are mainly C-C and C-H units. After treatment with phosphoric acid, the original compact crystalline structure of MCC became looser, exposing more and more glucose rings. In the C_1S_ XPS spectra of untreated and treated MCC, it is can thus be seen that the relative peak area of C_2_ (C-OH) decreased extensively (from 68.1% to 58.9% and to 56.3%), indicating that the hydrogen bond was destroyed after the phosphoric acid treatment. Moreover, it can be seen (Table 4) that the relative peak area of C_1_ (C-C/C-H) increased from 14.5% to 28.2% and to 33.1% with the decreasing of crystallinity, indicating that the hydrogen bond’s binding ability for cellulose chain was suppressed after the treatment of phosphoric acid, and so the intensity of C-C/C-H was increased.

**Table 4:** The C_1s_ XPS datum of MCC with different crystalline degree.

Xc %	Peak position			Separation			Area		
	*E*B/eV			*E*B/eV			*A*/%		
	C_1_	C_2_	C_3_	C_1_	C_2_	C_3_	C_1_	C_2_	C_3_
92.23	283.00	284.77	286.23	0.00	1.77	3.23	14.5	68.1	16.4
71.73	283.42	284.70	286.00	0.00	1.28	2.58	28.2	58.9	12.9
67.47	283.40	284.66	285.73	0.00	1.26	2.33	33.1	56.3	10.6

### 2.4. The kinetic analysis of cellulose dissolution in phosphoric acid

**Figure 8:**
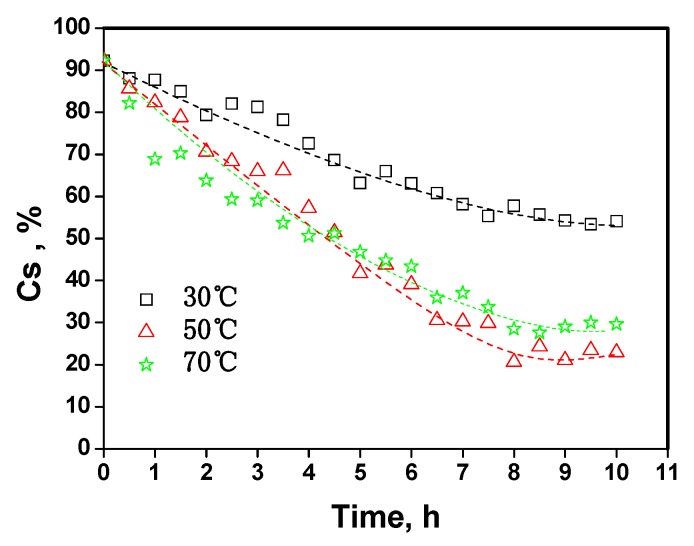
The relative crystalline cellulose content (Cs, which was determined by the intensity of 002 peak of sample’s XRD) in MCC with phosphoric acid.

The kinetics for MCC decrystallization was examined in a water bath. Figure 8 shows an example of the time-dependent decrystallization curves at three different temperatures over 10 h. The relative content of crystalline cellulose (Cs) in MCC decreased with time at all temperatures. At a temperature of 30 °C, the contents of crystalline cellulose reduced slowly and gradually leveled off at the end of reaction. The results indicated that the higher temperatures increased the rate of MCC decrystallization and resulted in lower proportions of crystalline cellulose. 

The kinetics for wheat straw decrystallization were derived under the assumption that the following conditions were met: (i) at reaction temperatures under 50 °C, the phosphoric acid did not lead to the decomposition of cellulose, hemicelluloses, and lignin in wheat straw, (ii) phosphoric acid only reacted with crystalline structures, (iii) the quality of phosphoric acid did not change during the reaction, and (iv) the crystallinity index measured by XRD represented the relative content of crystalline cellulose in MCC. We formulated an equation that provided a good description of the kinetics of the decrystallization behavior of phosphoric acid on MCC. The kinetic model for the decrystallization process is:

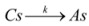
(1)
where *Cs* is the crystalline cellulose in MCC, *As* is the amorphous cellulose in MCC, and *Κ* is the decrystallization rate of MCC treated with phosphoric acid.

Based on this kinetic model, the kinetics for MCC decrystallization over the reaction time can be expressed with the following equation:

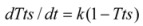
(2)
where *Tts* is the the fraction of crystalline cellulose in MCC. 

From equation (2), the following equation was derived:

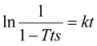
(3)


**Figure 9:**
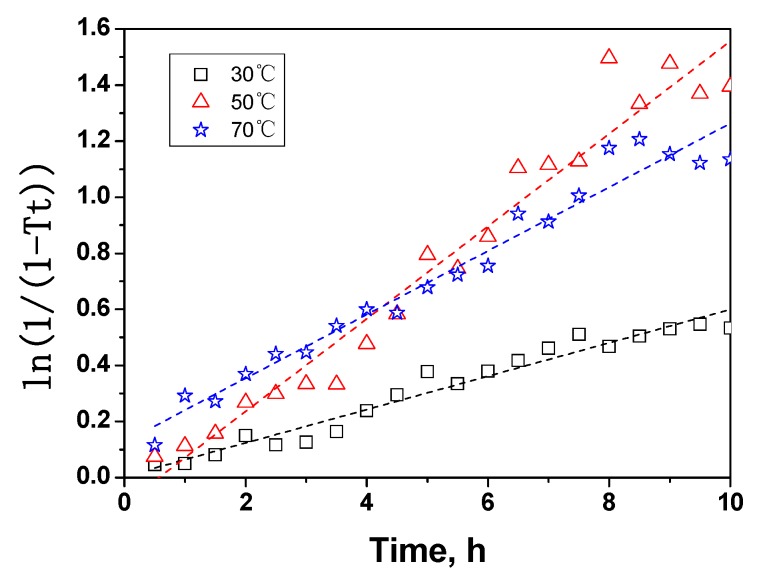
Transformation rate mathematical modeling of crystalline cellulose in MCC.

The conversion rates (*k*) of crystalline cellulose to amorphous cellulose at 30, 50, and 70 °C were obtained for equation (3) from the plots of the decrystallization experiments (Figure 9). 

The rate constants of decrystallization reaction at 30, 50, and 70 °C were 0.06, 0.17, and 0.12 h^-1^, respectively. The rate constants at 50 °C and 70 °C were much higher than that of 30 °C, thus higher temperature accelerated decrystallization reaction, but the constant at 70 °C was lower than that of 50 °C, indicating that acid hydrolysis was strong at the high temperature of 70 °C. In this paper, the kinetic equations were obtained by ignoring acid hydrolysis, therefore the kinetic equation at 70 °C was not well fitted. Based on the above observations, the temperature of 50 °C was deemed an optimal condition for cellulose dissolution in phosphoric acid. The apparent activation energies (Table 5) were calculated with the following Arrhenius equation [33]:

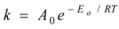
(4)


**Table 5:** Kinetics and Arrhenius parameters for the decrystallization of MCC with phosphoric acid.

Parameters	30 °C	50 °C	70 °C
*K/*h^-1^	0.06	0.17	0.12
*A_0_*_/_h^-1^	1.2 × 10^6^		
*Ea/*KJ·mol^-1^	42.4		

The activation energy of microcrystalline cellulose dissolving in phosphoric acid (83%) was 42.4 kJ/mol. Haisong Qi *et al*. [34] reported that the activation energy of cotton linters pulp dissolving in NaOH/urea system was about 101 kJ/mol, which was far bigger than *E_a_* in this paper. This suggests that phosphoric acid possess powerful solubilization towards cellulose. By combining equations (3) and (4), we obtained a modified prediction model that could be expressed as:

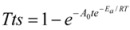
(5)


**Figure 10:**
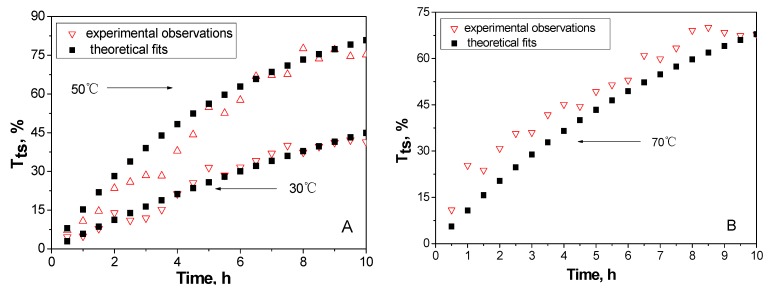
Experimental and mathematical transformation rate points of MCC: A (30 and 50 °C), B (70 °C).

To test the reliability of Equation (5), theoretical fits to the data obtained at 30 and 50 °C were compared to experimental observations (Figure 10).

Figure 10A shows the experimental and mathematical transformation rate of MCC at different temperatures. At the temperatures of 30 °C and 50 °C, the mathematical points agreed well with the experimental points , but at 70 °C (Figure 10B), the mathematical points deviated from the experimental points at a certain extent. This proved again that too high a temperature would cause acid hydrolysis and the mass loss of cellulose. The results indicated that the time-dependent conversion rate of MCC crystalline cellulose to amorphous cellulose could be predicted at high accuracy with the kinetic parameters determined from the Arrhenius equation.

## 3. Experimental

### 3.1. Materials

Microcrystalline Cellulose (MCC) was purchased from Shanghai Hengxin Chemical Reagent Co., Ltd. Phosphoric acid (83%) was obtained from Guangzhou Donghong Chemical Plant (China).

### 3.2. Sample preparation

First, MCC (1.0 g) was soaked in deionized water (1.0 mL) in a 50 mL beaker. Then, phosphoric acid (10 mL, 83%) was slowly added while agitating the beaker. The mixture was heated at 30, 50, or 70 °C for 10 h in a water bath, and a sample was collected each 0.5 h. Then, deionized water (50 mL) was added and the mixture was agitated vigorously. Precipitates formed immediately and they were collected with centrifugation. These samples were washed sequentially with ethanol (25 mL, twice) and deionized water (50 mL, twice), then neutralized with NaOH at pH 7.0, and finally dehydrated with acetone. Samples were dried at 50 °C overnight and then ground into powder. These samples were dried under vacuum with P_2_O_5_ desiccation in preparation for characterization. 

We have not identified the degradation products during the acid hydrolysis with high temperature. The cellulose products are still white.

### 3.3. X-ray diffraction method (XRD)

XRD measurements were performed on a Rigaku powder diffractometer (Rigaku Industrial Corporation, Japan) with CuKa radiation. The tube voltage was set at 40 kV, and the current was set at 30 mA with a wavelength of 0.1542 nm. The XRD diffraction patterns were taken over 2 h in the range of 5° to 50° at a scan speed of 12°/min, the step size was 0.02°, and the exposure time was 10 min. The results were treated with origin 8 to separate the peaks. The crystallinity index (*χ_c_*) was calculated with the following formula [35,36]:

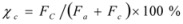



where, *F_c_* and *F_a_* are the area of the crystal (peak of cellulose I at 2θ=22.8°) and non-crystal regions (peak at 2θ=19.8°), respectively. 

### 3.4. Solid-State Cross-Polarization Magic Angle Spinning (CP/MAS) ^13^C-NMR

The solid-state CP/MAS ^13^C NMR spectra were obtained on a Bruker DRX-400 spectrometer (Bruker BioSpin Group) with a 5 mm MAS BBO probe that employed both cross-polarization and magic angle spinning. Each experiment was conducted at ambient temperature (20 ± 1 °C). The spectrometer was operated at 100 MHz. Acquisition time was 0.034 s, the delay time 2 s, and the proton 90°pulse time 4.85 s. Each spectrum represents an accumulation of 5,000 scans. The results were treated with Origin 8 to separate the peaks.

### 3.5. X-ray Photoelectron Spectroscopy (XPS)

All XPS studies were carried out using a Thermo-VG Scientific ESCALAB 250 photoelectron spectrometer (Thermo Fisher Scientific). The spectrometer was equipped with a monochromatic Al Kα X-ray source (hν = 1486.6 eV) at 500 m spot size of 150 W operating at 15 kV. All recorded peaks were corrected for electrostatic by setting the component peak of the saturated hydrocarbons in C_1S_ spectrum to 284.60 eV. In all experiments, the base pressure in the analysis chamber was less than 2 × 10^-9^ mbar.

## 4. Conclusions

The degree of crystallinity of cellulose treated with phosphoric acid decreased markedly. XRD analysis showed that the crystalline diffraction peaks decreased significantly. The high temperature accelerated the rate of cellulose dissolution in phosphoric acid, the side-reaction of acid hydrolysis was strong and caused a mass loss of cellulose at temperatures over 50 °C. At relatively low temperatures, the kinetic behaviors of the crystalline cellulose decrease rate in phosphoric acid obeyed the kinetic equation ln(1/1-T*ts*) well, with the rate constants of the decrystallization reactions at the temperatures of 30, 50, and 70 °C being 0.06, 0.17, and 0.12 h^-1^; the activation energy was 42.4 kJ/mol.
